# Obesity-Related Changes in Growth Hormone Stimulation Test Performance Under Pediatric Growth Hormone Deficiency

**DOI:** 10.3390/children13020299

**Published:** 2026-02-21

**Authors:** Semine Ozdemir Dilek, Fatma Özgüç Comlek

**Affiliations:** 1 Department of Pediatric Endocrinology, University of Health Sciences, Adana City Training and Research Hospital, Adana 01370, Turkey; 2 Department of Pediatric Endocrinology, Medical Faculty, Selçuk University, Konya 42250, Turkey; fatma.ozguc@selcuk.edu.tr

**Keywords:** growth hormone deficiency, clonidine stimulation test, overweight, obesity, Insulin-like growth factor 1, Growth velocity

## Abstract

**Highlights:**

**What are the main findings?**

- Obesity substantially impairs the diagnostic specificity of the clonidine stimulation test, leading to a marked increase in false-positive growth hormone deficiency diagnoses.
- A structured two-step algorithm integrating low GH peak thresholds with IGF-1 SDS and growth velocity restores diagnostic discrimination and significantly reduces obesity-related misclassification.

**What are the implications of the main findings?**

- Fixed GH cutoffs applied to clonidine stimulation testing are insufficient in children with obesity and risk inappropriate growth hormone treatment.
- A BMI-aware, auxology-integrated diagnostic strategy represents a more accurate and clinically sustainable framework for evaluating suspected GHD in contemporary pediatric populations.

**Abstract:**

Background/Objectives: The objective of this study is to determine the extent to which obesity alters the diagnostic reliability of the clonidine stimulation test (CST) for growth hormone deficiency (GHD) and whether incorporating insulin-like growth factor 1 (IGF-1) and the annual growth velocity standard deviation score (GV SDS) improves diagnostic precision. Methods: This retrospective study included 101 children evaluated for short stature using the clonidine stimulation test, with serum GH concentrations determined by a two-site, solid-phase, enzyme-labeled chemiluminescent immunometric assay (Immulite 2000 XPi, Siemens Healthcare Diagnostics, USA). Diagnostic performance was compared between overweight/obese (*n* = 47) and normal-weight (*n* = 54) groups. A two-step algorithm was evaluated: Step 1 applied a GH peak threshold of <5 ng/mL; Step 2 integrated IGF-1 SDS < −1.5 and annual GV SDS < −2.0 among children with subthreshold GH responses. Results: The median GH peak was significantly lower in overweight/obese children (4.5 [IQR 2.0–7.4] vs. 8.2 [5.1–11.5] ng/mL; *p* = 0.043). Although sensitivity remained comparable (82.6% vs. 90.5%; *p* = 0.666), elevated BMI markedly reduced specificity (50.0% vs. 84.8%; *p* = 0.008) and overall accuracy (66.0% vs. 87.0%; *p* = 0.017). Overweight/obese children demonstrated a higher proportion of false-positive CST results than non-obese children (25.5% vs. 9.3%). Among obese children with a GH peak of <5 ng/mL *(n* = 31), Step 2, which integrates IGF-1 and GV, improved specificity from 50% to 75% and the positive predictive value from 61.3% to 84.2%, correctly reclassifying 9 of 12 children without GHD who would otherwise have been misdiagnosed based on CST alone. Conclusions: Fixed GH cutoffs may lead to the misclassification of GHD in children with elevated BMI. Obesity significantly reduces the specificity and diagnostic accuracy of CST, increasing false-positive results. A two-step approach integrating IGF-1 and GV improves diagnostic precision and helps to differentiate true GHD from obesity-related GH suppression.

## 1. Introduction

Short stature is among the most common indications for referral to pediatric endocrinology and frequently necessitates a systematic diagnostic evaluation to determine potential underlying pathology [1]. The clonidine stimulation test (CST) remains a widely utilized first-line modality for diagnosing pediatric growth hormone deficiency (GHD), owing to its favorable safety profile and practical feasibility [2]. Despite its widespread use, the interpretation of growth hormone stimulation test (GHST) results is complicated by substantial heterogeneity in assay methodologies and the lack of a universally accepted GH peak cutoff value [3,4,5]. These limitations pose a risk of both over- and underdiagnosis of GHD, potentially affecting timely therapeutic intervention.

Modern chemiluminescent immunoassays (CLIAs) yield systematically lower GH concentrations compared with older radioimmunoassay methods [2]. To account for this assay-related difference, recent guidelines and studies have proposed lowering diagnostic thresholds from the historical 10 ng/mL to 7 ng/mL or even 5 ng/mL range, depending on local assay calibration and patient characteristics [2,6,7]. These differences are attributed to methodological heterogeneity in GH assays, as well as population-specific factors [3,6,7]. An observational study reported notable discrepancies between biochemical GH peaks and longitudinal growth trajectories, with discordance rates ranging from 6% to 42% depending on the applied cutoff values, highlighting the limitations of fixed thresholds and raising concerns about their predictive validity in diagnosing GHD [8].

A particularly relevant challenge arises in overweight and obese children, where GH responses are physiologically attenuated [9,10]. The blunted GH response commonly observed in overweight or obese children, despite adequate linear growth, may explain the discordance between biochemical stimulation test results and longitudinal growth outcomes, thereby underscoring the limited predictive validity of non-individualized diagnostic thresholds and reinforcing the need for phenotype-specific cutoffs in this population [10,11].

To address this challenge, we re-evaluated children with short stature from a cohort with a high prevalence of obesity who underwent clonidine GH stimulation testing and were subsequently followed for at least one year without receiving growth hormone therapy, allowing the assessment of spontaneous longitudinal growth patterns during the untreated period. We assessed the performance of a two-step diagnostic algorithm to refine the interpretation of clonidine stimulation test results and reduce obesity-related misclassification. In the first step, a stringent screening threshold was applied using a clonidine stimulation test (CST) GH peak of <5 ng/mL [2,12]. In the second step, functional markers of GH axis activity were incorporated, including an insulin-like growth factor-1 (IGF-1) standard deviation score (SDS) below −1.5 and an annual growth velocity SDS below −2.0 [13,14,15,16]. This study aimed to assess whether obesity leads to false-positive clonidine stimulation test results and whether a two-step diagnostic algorithm improves diagnostic accuracy.

## 2. Material and Methods

### 2.1. Study Design and Participants

This retrospective cohort study included 101 children aged 5–14 years who underwent GH stimulation testing with clonidine between 2020 and 2022 at a secondary care center with a pediatric endocrinology unit and who had not received prior growth hormone therapy. The test was performed in accordance with standard clinical indications for suspected GHD based on international guidelines [2].

The inclusion criteria were as follows: (1) height standard deviation score (SDS) < −2 for age and sex [17]; (2) height > 1.5 SDS below mid-parental target height; and (3) performance of a clonidine stimulation test, followed by subsequent re-evaluation including auxological and confirmatory biochemical assessments [18].

Exclusion criteria comprised chronic systemic illness (e.g., renal, gastrointestinal, or inflammatory conditions), previous or ongoing treatments known to affect the GH–IGF-1 axis (e.g., corticosteroids and chemotherapy), endocrine disorders (e.g., central hypothyroidism, precocious puberty, and delay in puberty), genetic syndromes (e.g., Turner, Noonan, and Prader–Willi), known intracranial pathology, malnutrition, small gestational age or preterm status without adequate catch-up growth, and incomplete or inconsistent data.

### 2.2. Anthropometric and Growth Assessment

All anthropometric measurements were obtained by trained personnel following standardized procedures. Body mass index (BMI) was calculated for all participants and categorized based on age- and sex-specific percentiles according to Neyzi et al. [17]. Those with a BMI ≥ 85th percentile and a <95th percentile were classified as overweight, and those with a BMI ≥ 95th percentile were classified as obese. Children with normal weight and those classified as overweight or obese were included in this study; patients with malnutrition were excluded. The annual GV SDS was calculated as the difference between two height measurements obtained over a one-year interval, and the height velocity SDS was determined using age- and sex-adjusted reference standards [19]. An annual GV SDS threshold of −2.0 was selected as a supportive auxological indicator within the diagnostic algorithm, consistent with established definitions of pathological growth failure [15,20].

Pubertal status was assessed using Tanner staging, with pubertal onset indicated by breast development at stage II or above in girls and testicular volume ≥ 4 mL (measured using a Prader orchidometer) in boys [21,22]. To minimize the confounding impact of pubertal maturation on stimulated GH secretion, the analytic cohort was limited to children in pre/early puberty (Tanner stages I–II) at the time of testing. Participants at Tanner stages III–V were excluded.

### 2.3. GH Stimulation Testing

All patients underwent clonidine stimulation testing as the initial diagnostic test. Clonidine (Catapres^®^, Boehringer Ingelheim, Ingelheim am Rhein, Germany) was administered orally at a dose of 0.15 mg/m^2^ (maximum 0.25 mg), and venous blood samples were obtained at baseline and at 30, 60, 90, and 120 min following drug administration [12]. Serum GH concentrations were measured using a two-site, solid-phase, enzyme-labeled chemiluminescent immunometric assay (Immulite 2000 XPi, Siemens Healthcare Diagnostics, Tarrytown, NY, USA), which was calibrated against WHO IS 98/574 (Analytical sensitivity: 0.01 ng/mL; intra-assay CV: 3.2–5.8%; inter-assay CV: 4.5–6.5%). The diagnostic classification of GHD in our study was based on the predefined threshold of 7 ng/mL, in accordance with the guidelines and the literature [2,5,12]. Among the 61 children with a CST GH peak of <7 ng/mL, a second GH stimulation test was performed on a separate day, with a minimum interval of three days [12]. Accordingly, 24 patients were evaluated using an insulin-tolerance test (ITT), and 37 patients were evaluated using the L-dopa test [2]. After an overnight fast, L-dopa (Madopar^®^, Roche, Basel, Switzerland) was administered (10 mg/kg; maximum 500 mg). The ITT was used to assess growth hormone (GH) secretion in children with suspected GH deficiency. Tests were performed in the morning after at least 8 h of fasting. Regular insulin (0.05–0.1 U/kg) was administered intravenously, and blood glucose was monitored at regular intervals. Adequate hypoglycemia was defined as a plasma glucose level of <40 mg/dL or a ≥50% decrease from baseline.

#### 2.3.1. Diagnostic Criteria

Growth hormone deficiency was diagnosed according to established criteria [3,4,17,18,19]:

1. Biochemical: GH peak < 7 ng/mL on both clonidine and L-dopa or ITTs.
2. Auxological: GV SDS < −2 over 1 year [15,20,23].
3. Supportive (not mandatory): Magnetic resonance imaging (MRI) findings consistent with hypothalamic–pituitary pathology (e.g., pituitary hypoplasia, ectopic posterior pituitary, or pituitary stalk abnormalities).

#### 2.3.2. False-Positive Classification

Participants were classified as false positives when initial clonidine stimulation test results suggested growth hormone deficiency, but auxological or secondary GHST results contradicted this diagnosis. False positives were defined as participants meeting both of the following criteria: GH peak < 7 ng/mL on the CST but GH peak ≥ 7 ng/mL on confirmatory testing (L-dopa or ITT) and annual GV SDS ≥ −2 during the 12-month untreated follow-up (indicating adequate spontaneous growth despite the initial low CST result).

#### 2.3.3. False-Negative Classification

**Figure 1:**
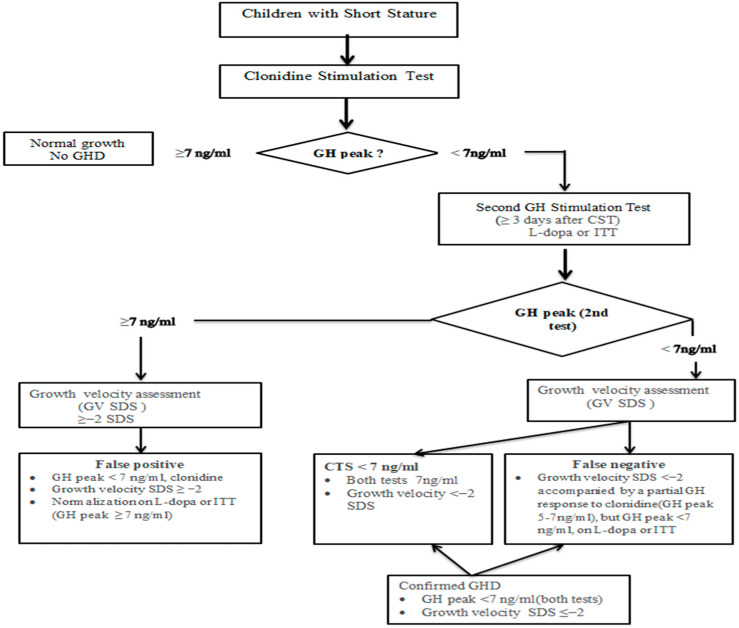
Diagnostic algorithm for evaluation of pediatric growth hormone deficiency. GHD: growth hormone deficiency; GH: growth hormone; GV SDS: growth velocity standard deviation score; CST: clonidine stimulation test. MRI findings (e.g., pituitary hypoplasia, ectopic posterior pituitary, or pituitary stalk abnormalities) were considered supportive information. Imaging was not mandatory and was not required for GHD diagnosis, which was confirmed based on the conventional 7 ng/mL criterion used in Table 1. The 5 ng/mL threshold was employed solely for exploratory index-test and integrated algorithm performance analyses, as shown in Table 2 and Table 3.

Participants were classified as false negatives when clinical indicators strongly supported GHD but biochemical testing yielded borderline CST results (growth velocity SDS < −2 or annual height gain < 4 cm (consistent with clinical GHD)), accompanied by a partial GH response to clonidine (GH peak: 5–7 ng/mL) but a GH peak of <7 ng/mL for L-dopa or the ITT. The diagnostic algorithm is shown in Figure 1.

**Table 1:** Diagnostic performance of the CST for confirmed GHD using the conventional 7 ng/mL reference standard, stratified by weight status.

Parameter	Overweight/Obese (*n* = 47)	Normal Weight (*n* = 54)	*p*-Value
GH peak, ng/mL, median (IQR)	4.5 (2.0–7.4)	8.2 (5.1–11.5)	0.043
Test Performance Metrics			
True positives	19 (40.5%)	19 (35.2%)	
False positives	12 (25.5%)	5 (9.3%)	
True negatives	12 (25.5%)	28 (51.5%)	
False negatives	4 (8.5%)	2 (4%)	

GH: growth hormone; CST: clonidine stimulation test.

**Table 2:** Diagnostic characteristics and test performance metrics for the clonidine stimulation test at the 5 ng/mL index threshold.

Diagnostic Characteristics			
Parameter	Overweight/Obese (*n* = 47)	Normal Weight (*n* = 54)	*p*-Value
GH peak < 5 ng/mL *n* (%)	31 (66)	24 (44.4)	0.049
Test performance metrics threshold 5 ng/mL % (95% CI)			
Sensitivity	82.6 (62.9–93.0)	90.5 (71.1–97.3)	0.666
Specificity	50 (31.4–68.6)	84.8 (79.1–93.3)	0.008
PPV	61.3 (43.8–76.3)	79.2 (59.5–90.8)	0.240
NPV	75 (50.5–89.8)	93.3 (78.7–98.2)	0.163
Positive LR	1.70 (1.15–2.51)	5.97 (2.63–13.55)	
Negative LR	0.35 (0.13–0.92)	0.11 (0.03–0.42)	
Accuracy	66.0 (51.2–77.8)	87.0 (75.6–93.6)	0.017

CI: confidence interval; PPV: positive predictive value; NPV: negative predictive value; LR: likelihood ratio; GH: growth hormone; Statistical tests: Continuous variables were compared using the Mann–Whitney U test. Categorical variables were analyzed using Pearson’s χ^2^ test or Fisher’s exact test, as appropriate. *p* < 0.05 was considered statistically significant.

**Table 3:** Performance of the integrated diagnostic algorithm in overweight/obese children (*n* = 47).

Algorithm Step	*n*	GHD n:23	NonGHD n:24	Sensitivity	Specificity	PPV	NPV
Step 1: Clonidine GHST peak							
GH ≥ 5 ng/mL → GHD excluded	16	4	12	82.6%	50%	61.3%	75%
GH < 5 ng/mL → Proceed to Step 2	31	19	12				
Step 2: IGF-1 SDS and GV SDS (among those with GH ≤ 5)							
IGF-1 SDS < −1.5 and GV SDS < −2.0 → High probability GHD → Confirmatory test	19	16	3	84.2%	75%	84.2%	75%
IGF-1 SDS ≥ −1.5 or GV SDS ≥ −2.0 → Likely related to elevated BMI → Weight management	12	3	9				

GHD: growth hormone deficiency; GH: growth hormone; IGF-1: insulin-like growth factor 1; GV: growth velocity; SDS: standard deviation score; PPV: positive predictive value; NPV: negative predictive value; LR^+^: positive likelihood ratio; LR^−^: negative likelihood ratio.

#### 2.3.4. IGF-1 Measurement and Categorization

Insulin-like growth factor 1 (IGF-1) was measured after acid–ethanol extraction (Immulite 2000 XPi, Siemens Healthcare Diagnostics, Tarrytown, NY, USA), which was calibrated against WHO NIBSC 1st IRR 87/518 (intra-assay CV: 2.3–3.9%; inter-assay CV: 3.8–8.2%). Values were standardized to the SDS using age- and sex-specific Turkish reference data [17]. Serum IGF-1 concentrations were measured at the baseline (0 min) of the clonidine stimulation test and used for subsequent analysis. The IGF-1 SDS threshold of −1.5 was incorporated as a supportive component of the diagnostic algorithm to aid discrimination between functional deficiency and true growth hormone deficiency [13,14,24]. Values were categorized as follows: IGF-1 SDS < −1.5 or IGF-1 SDS ≥ −1.5. The cutoff values used in the methodological approach were defined according to Figure 1.

#### 2.3.5. Index Test: Two-Step Diagnostic Algorithm

To address potential diagnostic misclassification related to obesity-associated attenuation of stimulated GH responses, we re-evaluated a two-step diagnostic algorithm within an index-test framework [10,25].

#### 2.3.6. Step 1—Biochemical Screening

In the first step, a clonidine-stimulated GH peak threshold of <5 ng/mL was applied as the index criterion [5,18,26]. Children with GH peak values ≥ 5 ng/mL were considered to have sufficient GH secretion within the screening framework and did not proceed to the second step. Those with peak values < 5 ng/mL underwent further auxological and biochemical assessment. Diagnostic accuracy measures for this threshold were calculated separately according to BMI status and are presented in Table 2.

It is important to emphasize that this threshold served as a screening criterion within the index-test framework and not as a standalone diagnostic definition of GHD. The reference standard for confirmed GHD remained as a GH peak of <7 ng/mL upon both clonidine testing and a second stimulation test (L-dopa or ITT), as defined in Section 2.3 and Table 1 (7 ng/mL).

#### 2.3.7. Step 2—Auxological and Biochemical Integration

Among children with a GH peak of <5 ng/mL, additional criteria were applied to differentiate probable GHD from physiological suppression associated with excess adiposity.

The following parameters were evaluated:

IGF-1 standard deviation score (SDS) < −1.5 [13,14,24].

Growth velocity (GV) SDS < −2.0 [15,20,23].

Children fulfilling both criteria were considered to have a high likelihood of GHD and were referred for confirmatory second GH stimulation testing. Children not meeting both criteria were considered more likely to have obesity-related GH suppression. The diagnostic performance of the integrated two-step approach is presented in Table 3.

#### 2.3.8. Rationale for Re-Evaluating the Lower Index Threshold

Assessment of the 5 ng/mL level was undertaken in light of the high prevalence of overweight and obesity in the study population and the recognized physiological blunting of stimulated GH responses associated with adiposity [5,6,25,26]. In addition, immunometric assays yield lower GH concentrations, and several reports have suggested that discriminatory capacity is greatest at lower peak values, while results between 5 and 10 ng/mL may overlap with normal secretion [18]. Therefore, the lower 5 ng/mL value was examined to evaluate test performance and the potential reduction in false-positive classifications [6,19].

#### 2.3.9. Magnetic Resonance Imaging Protocol

A pituitary MRI protocol was applied to 52 patients. Imaging was performed using a 1.5-Tesla scanner (Siemens, Erlangen, Germany). The protocol included sagittal T1-weighted and coronal T2-weighted sequences with a slice thickness of 3 mm. Pituitary morphology was evaluated and categorized as normal, hypoplastic, ectopic posterior pituitary, or pituitary stalk abnormality [2].

### 2.4. Statistical Analysis

All statistical analyses were performed using SPSS Statistics version 26.0 (IBM Corp., Armonk, NY, USA) and MedCalc Statistical Software version 20.215 (MedCalc Software Ltd., Ostend, Belgium). The normality of continuous variables was assessed using the Shapiro–Wilk test. Normally distributed continuous variables are presented as the mean ± standard deviation (SD) and compared using independent-sample t-tests, whereas non-normally distributed variables are expressed as the median (interquartile range, IQR) and compared using the Mann–Whitney U test. Categorical variables were analyzed using Pearson’s chi-square or Fisher’s exact test, as appropriate.

Diagnostic performance metrics including sensitivity, specificity, the positive predictive value (PPV), the negative predictive value (NPV), likelihood ratios (LR^+^ and LR^−^), and accuracy were calculated for each step of the diagnostic algorithm, using confirmed GHD as the reference standard. Ninety-five percent confidence intervals (95% CIs) for proportions were estimated using the Wilson score method. Comparisons between proportions (e.g., Step 1 vs. Step 2) were performed using Pearson’s chi-square or Fisher’s exact test. Receiver operating characteristic (ROC) curves were constructed to assess discriminatory performance, and the areas under the curves (AUCs) were compared using DeLong’s test. A two-sided *p*-value < 0.05 was considered statistically significant.

## 3. Results

A total of 101 children (54.5% male; mean age: 10.9 ± 2.6 years) were included. Puberty was present in 37.6%. GHD was diagnosed in 43.6%, and 46.5% were classified as obese/overweight. According to BMI percentiles, 53.5% of the children were classified as normal weight, 12.9% as overweight, and 33.7% as obese. The participants comprised 63 children (62.4%) at Tanner stage I and 38 children (37.6%) at Tanner stage II. The distribution of pubertal status did not differ significantly between sexes: 63.6% of males (35/55) and 60.9% of females (28/46) were prepubertal (*p* = 0.771). GH peak values differed according to pubertal stage. However, the median GH peak did not differ significantly between Tanner stages 1 and 2 (6.8 vs. 7.1 ng/mL, *p* = 0.512) (Table 4).

When stratified by weight category, the median GH peak demonstrated a progressive decline (8.2 ng/mL; IQR: 5.1–11.5) in normal-weight children, (5.9 ng/mL; IQR: 3.5–8.1) in overweight children, and (3.6 ng/mL; IQR: 1.8–6.0) in obese children—suggesting a dose–response relationship between adiposity and GH suppression. Correspondingly, the proportion with a GH peak of <5 ng/mL increased from 44.4% (24/54) to 53.8% (7/13) to 70.6% (24/34) across these groups, respectively.

The median GH peak levels were significantly lower in overweight/obese children [4.5 (2.0–7.4) ng/mL] than in those with normal weight [8.2 (5.1–11.5) ng/mL] (*p* = 0.043). False-positive CST results were markedly more frequent in the overweight/obese group (12/47, 25.5%) than in the normal-weight group (5/54, 9.3%) (Table 1).

The diagnostic performance metrics of the clonidine stimulation test at the 5 ng/mL threshold are summarized in Table 2. Among participants with a GH peak of <5 ng/mL, the frequency was significantly higher in the overweight/obese group (66%, 31/47) than in the normal-weight group (44.4%, 24/54) (*p* = 0.049). Sensitivity did not differ significantly between the overweight/obese and normal-weight groups (82.6% vs. 90.5%; *p* = 0.666). In contrast, specificity was significantly lower in the overweight/obese group (50.0% vs. 84.8%; *p* = 0.008). Both the positive predictive value (PPV) and the negative predictive value (NPV) tended to be lower among overweight/obese individuals (PPV: 61.3% vs. 79.2%, *p* = 0.240; NPV: 75.0% vs. 93.3%, *p* = 0.163), although these differences did not reach statistical significance. The positive likelihood ratio was markedly reduced in obese patients (LR^+^: 1.70 vs. 5.97), as was the negative likelihood ratio (LR^−^: 0.35 vs. 0.11). Overall diagnostic accuracy was significantly higher in the normal-weight group (87.0%, 95% CI: 75.6–93.6) compared with the obese/overweight group (66.0%, 95% CI: 51.2–77.8; *p* = 0.017).

A structured two-step diagnostic algorithm was evaluated to enhance the diagnostic accuracy of growth hormone deficiency (GHD) and reduce obesity-related false-positive results. In Step 1, the application of a clonidine-stimulated GH peak threshold of <5 ng/mL correctly identified 19 of 23 patients with confirmed GHD, yielding a sensitivity of 82.6%, but demonstrated limited specificity (50%), with a positive predictive value (PPV) of 61.3% and a negative predictive value (NPV) of 75%. In Step 2, the 31 patients with a GH peak of <5 ng/mL were further assessed using the combined criterion of IGF-1 < –1.5 SDS and GV < −2.0 SDS; this markedly improved diagnostic discrimination, accurately identifying 16 of 19 confirmed GHD cases and misclassifying only three non-GHD subjects. The outcome was a sensitivity of 84.2%, a specificity of 75%, a PPV of 84.2%, and an NPV of 75%. Conversely, patients with IGF-1 ≥ −1.5 SDS or GV ≥ −2.0 SDS were predominantly non-GHD (9/12) (Table 3).

Pituitary MRI abnormalities were detected in eight of 52 patients (15.4%): pituitary hypoplasia in four, ectopic posterior pituitary in two, and pituitary stalk interruption in two.

## 4. Discussion

Interpreting GH stimulation test results in children remains one of the most debated areas of pediatric endocrinology. The diagnostic landscape has evolved substantially over the past decade, driven by advances in assay technology, the increasing prevalence of pediatric obesity, and accumulating evidence that fixed biochemical thresholds inadequately reflect the physiologic complexity of GH-IGF-1 axis function, prompting redefinition of diagnostic cutoffs [2,27]. Historically, GHSTs were adopted to differentiate physiological variation from true GHD, yet their ability to reflect biologically meaningful GH–IGF-1 axis function has been increasingly questioned [3,4,6,28]. This study evaluated the diagnostic performance of the clonidine stimulation test as an initial screening tool, not as a standalone diagnostic criterion. Our findings contribute to this ongoing re-evaluation by quantifying the extent to which overweight/obesity influences GH responses and by assessing a practical, two-step diagnostic algorithm designed to mitigate misclassification. Our findings suggest that, consistent with current recommendations, a single CST, given its limited reproducibility and high false-positive rates, has restricted value as an initial screening tool in children with obesity, while the interpretation of borderline or low responses may be enhanced by integrating growth velocity and IGF-1 data within the clinical context [2,5].

Modern immunometric assays yield lower GH peak values than older radioimmunoassay methods, raising concerns that outdated higher cutoffs may lead to false-positive diagnoses [29,30]. Emerging evidence, including proposed thresholds in the 3–5 ng/mL range, supports recalibrating diagnostic criteria to align with contemporary assay performance and reduce misclassification of GH-sufficient children [5]. Our data reflect this pattern, as a 5 ng/mL threshold provided high sensitivity yet limited specificity, particularly in children with obesity. These findings underscore the need for assay-specific and context-appropriate GH cutoffs to improve the diagnostic performance of clonidine-stimulated testing [6,7,31]. The selection of a clonidine-stimulated GH index test cutoff of 5 ng/mL was based on evidence indicating that the discriminative value of provocative testing is concentrated at lower peak levels [29]. Registry data show that children with GH peaks between 5 and 10 ng/mL are largely indistinguishable from those with normal secretion, and clonidine is known to elicit higher GH responses than other provocative agents [29,32]. Borges et al. [24] identified an optimal CST threshold of approximately 3 ng/mL, noting that higher cutoffs substantially increased false-positive rates, particularly in populations with a high prevalence of obesity. This approach is further supported by the limited inter-test correlation of GH responses (r = 0.35–0.60), accounting for only 12–36% of total variability, underscoring the need for stimulus- and assay-specific interpretation rather than universal cutoffs [28]. In our cohort, GH peak values were similar across Tanner stages, and we did not detect a significant difference. While puberty is known to affect GH secretion, this finding suggests that pubertal status by itself was probably not a major factor influencing CST results in our group.

Beyond methodological variability, obesity markedly complicates the interpretation of GH stimulation tests, as excess adiposity blunts both spontaneous and stimulated GH secretion through mechanisms involving insulin resistance, elevated free fatty acids, and altered somatostatin tone, leading to functional rather than pathological suppression [11]. Consistent with this physiology, overweight/obese children in our cohort exhibited significantly lower peak GH responses compared with their normal-weight peers, a pattern well documented in prior studies [11,31]. Evidence from previous studies reinforces that this physiologic pattern increases the risk of overdiagnosis in populations with a high prevalence of obesity [9,10]. Our findings extend these observations by demonstrating their clinical implications: Although obese children were substantially more likely to produce GH peaks < 5 ng/mL, confirmatory evaluation revealed that only a minority truly had GHD. This discordance indicates that low GH peaks in obesity often represent physiological suppression rather than genuine endocrine insufficiency. In our cohort, 25.5% of children with elevated BMI and subthreshold GH responses were ultimately misclassified as having false-positive GHD based on CST results alone. This rate was nearly threefold higher than that observed in normal-weight peers (9.3%; *p* = 0.025). Children in this group would have met conventional biochemical criteria for GH replacement despite retaining adequate endogenous GH secretion. Taken together, these findings underscore that CST performance is strongly modulated by BMI status and that a fixed GH threshold cannot adequately distinguish physiological suppression from pathological deficiency [5]. Inaccurate interpretation of the GHST and inappropriate clinical decision-making would therefore have led to unnecessary treatment, avoidable costs, and a cascade of follow-up decisions anchored in a misclassification [33,34]. Despite growing evidence in favor of lower and BMI-adjusted GH cutoffs for overweight or obese children, a consensus obesity-specific threshold has yet to be established in pediatric practice [7,11,25].

Although stimulated GH secretion is attenuated in pediatric obesity, hepatic GH sensitivity and basal IGF-1 production remain relatively preserved, contributing to the well-described GH–IGF-1 discordance in this population [27]. Children who maintain adequate growth velocity despite low GH peaks demonstrate preserved GH–IGF axis function, even if stimulation test results indicate the contrary. Prior studies similarly suggest that normal growth in the setting of low peak GH values predominantly represents physiological variation rather than true endocrine deficiency [16,35].

In light of these considerations, we evaluated a two-step diagnostic algorithm designed to incorporate physiologically grounded markers into CST interpretation. The first step, utilizing a GH cutoff of <5 ng/mL, provided strong sensitivity but poor specificity. The addition of IGF-1 SDS and GV SDS in the second step markedly strengthened diagnostic accuracy by incorporating indicators of GHD. This refinement improved specificity from 50% to 75% while maintaining high sensitivity, demonstrating that an integrated biochemical–auxological approach can effectively distinguish true GHD from obesity-related suppression. The justification for the use of stringent thresholds for IGF-1 SDS (<−1.5) and GV SDS (<−2.0) is significant in the context of the high obesity prevalence in our cohort, as a GV cutoff of −2.0 SDS served as a safeguard against misclassifying children with obesity-related physiological GH suppression as having true GHD [15,20]. By excluding milder forms of GHD, this stringent GV threshold reduced the risk of misclassifying transient growth deceleration associated with obesity as true GHD, thereby minimizing unnecessary treatment exposure [33,36]. The IGF-1 SDS threshold of −1.5 represents a literature-supported intermediate criterion [13]. Pediatric ROC-based studies have consistently identified thresholds in the range of −1.5 to −2.0 SDS as optimizing the balance between sensitivity and negative predictive values for GHD diagnosis [13,14,24]. Importantly, both parameters were applied as supportive elements within a comprehensive diagnostic framework rather than as standalone exclusion criteria, in accordance with current guideline recommendations [12].

Also consistent with current recommendations, clonidine stimulation testing alone was not used for diagnosis but served to illustrate the potential for misclassification at the initial screening stage, which was substantially reduced using the second-step approach [2]. The susceptibility of GH stimulation tests to false-positive results driven by the inherent biological variability of pharmacologic stimuli and modulating factors highlights the discordance that can emerge between the pharmacologically induced GH response and the underlying somatotropic function. This discordance provides a strong rationale for adopting a structured, multi-step diagnostic strategy that integrates both biochemical and auxological indicators. Our findings complement current guidelines advocating for combined biochemical and auxological evaluation, yet they also extend prior work by offering a clear operational pathway tailored to the diagnostic challenges encountered in obese children. While current guidelines emphasize integrating multiple diagnostic parameters, our approach provides a structured and clinically applicable framework for interpreting biochemical and longitudinal markers of growth hormone axis activity in children with obesity [12]. This approach also reduces unnecessary GH treatment and minimizes both potential risks and healthcare costs [3,37].

These findings are consistent with the emerging literature that increasingly supports consideration of center-specific, assay-calibrated, and BMI-informed interpretive approaches alongside traditional threshold-based criteria [29,30]. In normal-weight children, CST retained reasonable diagnostic value, aligning with traditional expectations. However, in children with obesity, accuracy declined sharply, necessitating a more comprehensive interpretive framework. These findings resonate with the growing literature supporting the transition from static, assay-independent thresholds to center-specific, assay-calibrated, and BMI-adjusted interpretive models that better reflect real-world diagnostic complexity.

Despite these strengths, our study has limitations. The retrospective design introduces the possibility of selection bias, and findings from a secondary care referral population may not be generalizable to community-based cohorts. The high prevalence of obesity in our cohort likely reflects both referral bias inherent to secondary care centers and broader regional population trends [38]. The sample size was determined by the number of available cases meeting the predefined inclusion criteria rather than by using a prospective power calculation. A post hoc analysis demonstrated adequate statistical power (>80%) to detect clinically meaningful differences in specificity between BMI groups. Nevertheless, larger multicenter studies would enhance generalizability and allow more definitive subgroup analyses. The present study analyzed overweight or obese children as a combined group rather than as distinct BMI categories. While it is plausible that increasing the degree of adiposity exerts differential effects on stimulated GH responses, the relatively small number of participants in the overweight subgroup prevented adequately powered stratified diagnostic accuracy analyses.

Future investigations involving larger cohorts in multicenter settings are warranted to determine whether BMI-specific thresholds or tailored algorithmic strategies across the adiposity spectrum may further improve diagnostic precision.

Nonetheless, our study provides practical insight into the evaluation of GHD by demonstrating that elevated BMI systematically influences clonidine stimulation test performance. Median GH peaks were lowest in children with obesity (3.6 ng/mL), intermediate in overweight individuals (5.9 ng/mL), and highest in normal-weight peers (8.2 ng/mL). Despite these lower responses, children with obesity had the greatest likelihood of false-positive classification, with approximately one in four showing subthreshold CST results in the absence of true GHD. These findings underscore the need for weight-adjusted interpretative strategies integrating growth velocity and IGF-1 alongside CST results.

## 5. Conclusions

Our findings indicate that elevated BMI is associated with reduced specificity and higher false-positive rates in clonidine stimulation testing. A two-step approach incorporating IGF-1 and growth velocity was associated with improved diagnostic discrimination between true GHD and obesity-related GH suppression. Taken together, these results suggest that BMI-informed, assay-specific interpretive strategies may be helpful in clinical practice, particularly in settings with a high prevalence of pediatric obesity.

## Figures and Tables

**Table 4:** Baseline demographic, clinical, and auxological characteristics of the study population.

	Category/Value	Frequency (*n*)	Percentage (%)	Mean ± SD	Median *p*-Value (Min–Max)
Gender					
Male	Male	55	54.50		
Female	Female	46	45.50		
Growth Hormone Deficiency (GHD)		44	43.6		
Normal Weight Overweight Obesity		54 13 34	53.5 12.9 33.7		
Age (years)				10.9 ± 2.6	
GH Peak (ng/mL)					5.1 (0.2–33)
Growth Velocity SDS (GV SDS)/year					−1.5 (−3.5–0.6)
					CST GH peak (ng/mL)
Pubertal Status	Tanner 2 Tanner 1	38 63	37.60 62.40		7.1 (0.5–16) 0.512 6.8 (0.2–33)
	Pubertal Status Tanner 2 Tanner 1	Male (*n*) 20 35	Female (*n*) 18 28		0.771

Data are presented as frequency (*n*) and percentages (%) for categorical variables and as the mean ± standard deviation (SD) or median (minimum–maximum) for continuous variables. GHD: growth hormone deficiency; GH: growth hormone; GV SDS: growth velocity standard deviation score; CST: clonidine stimulation test. Obesity/overweight was classified based on age- and sex-specific BMI percentiles according to national growth charts.

## Data Availability

The datasets generated and/or analyzed in this study are available from the corresponding author on reasonable request due to privacy, legal, and ethical reasons.
